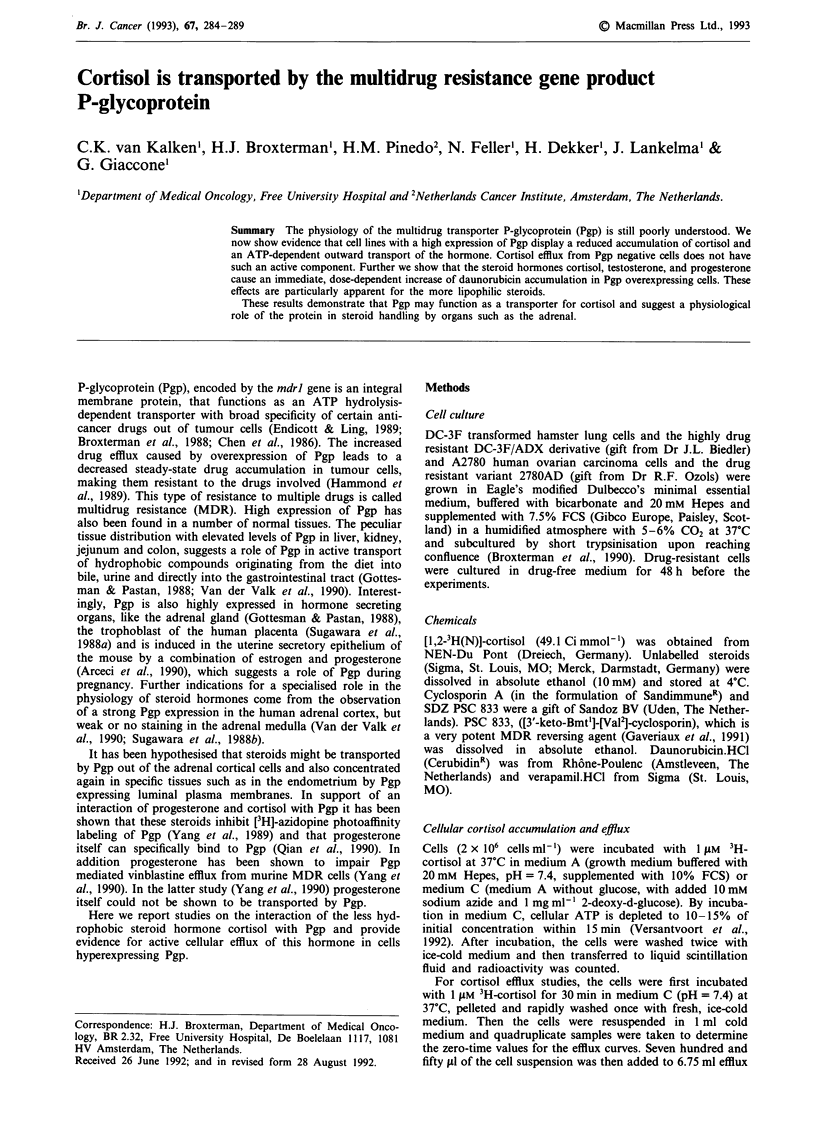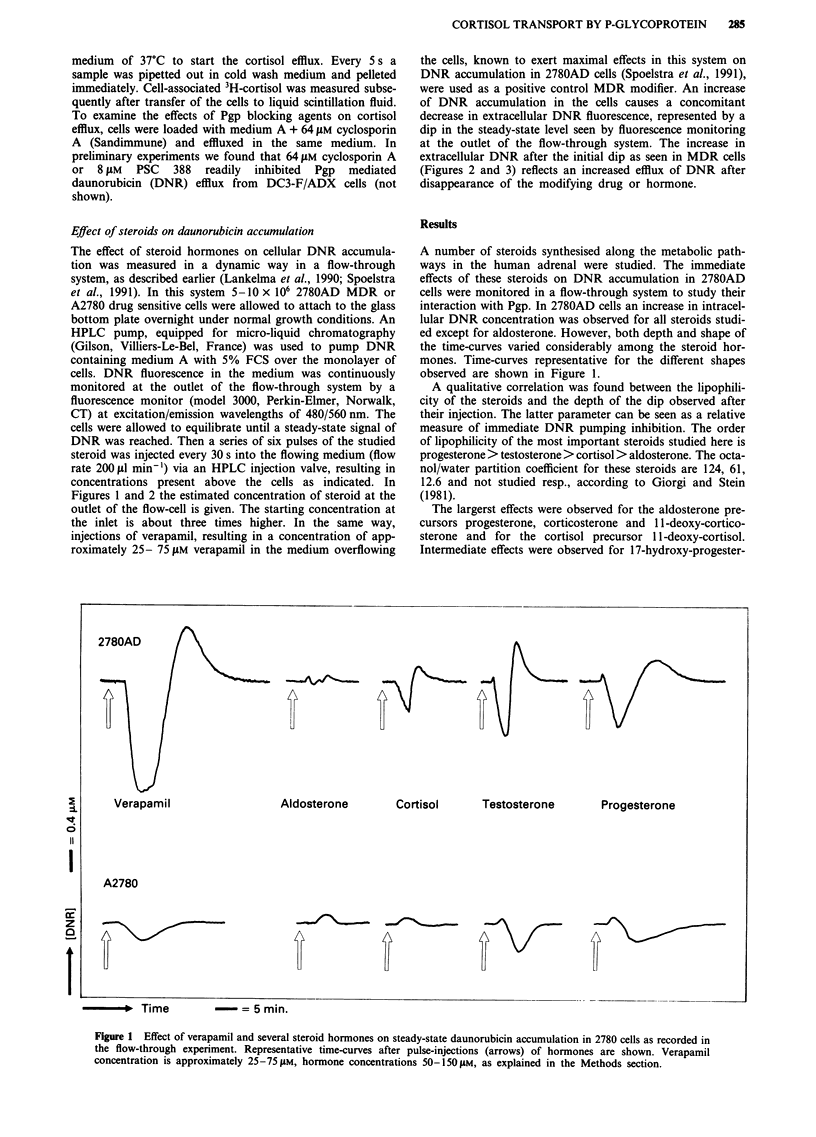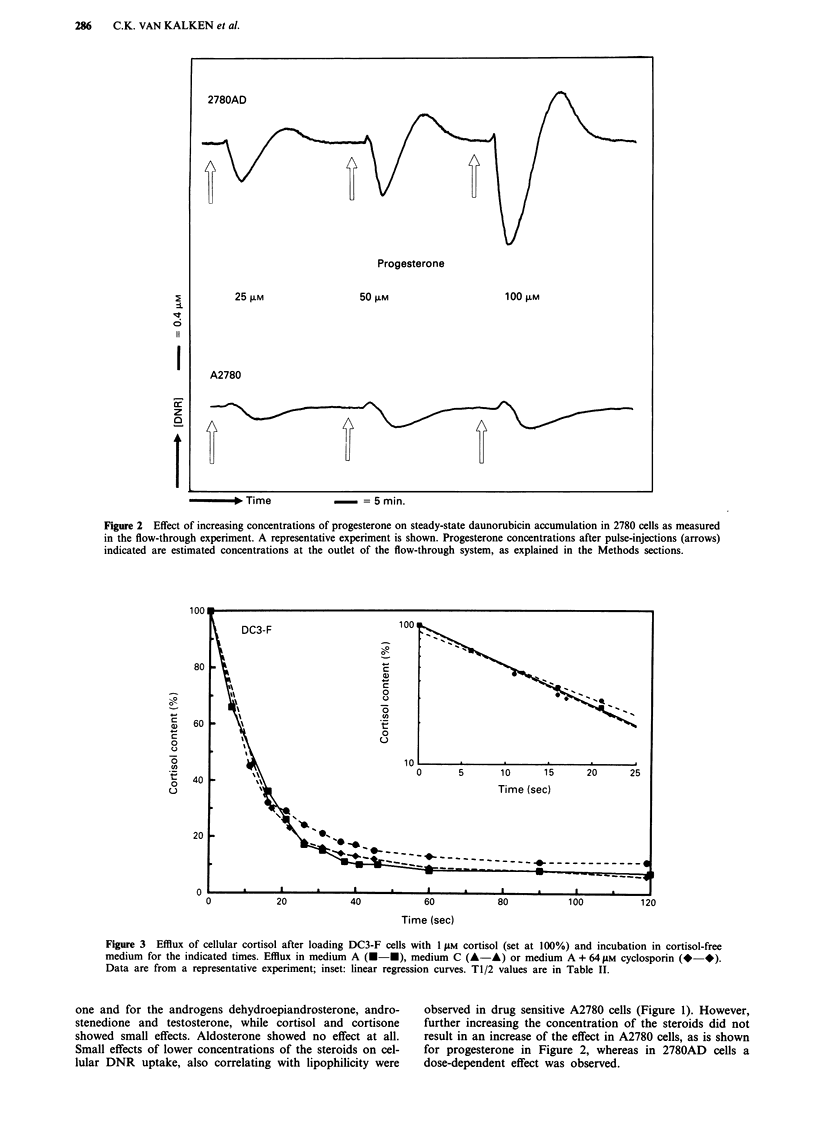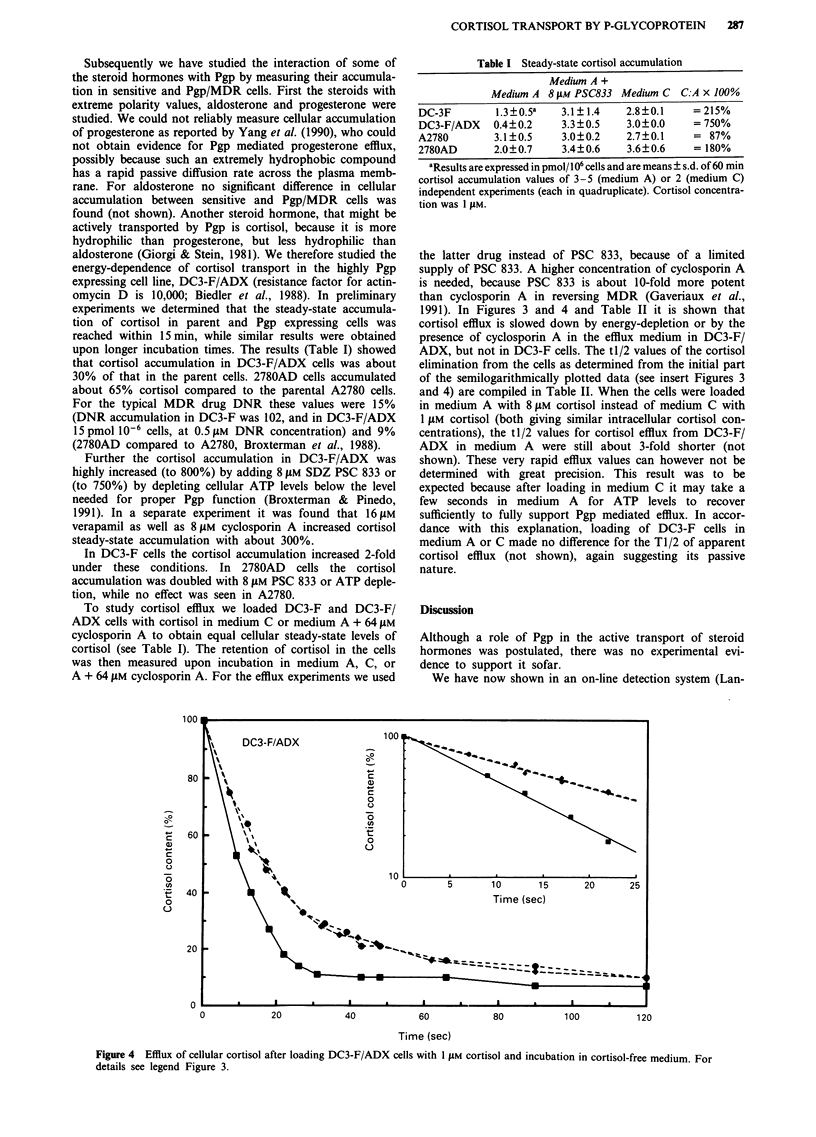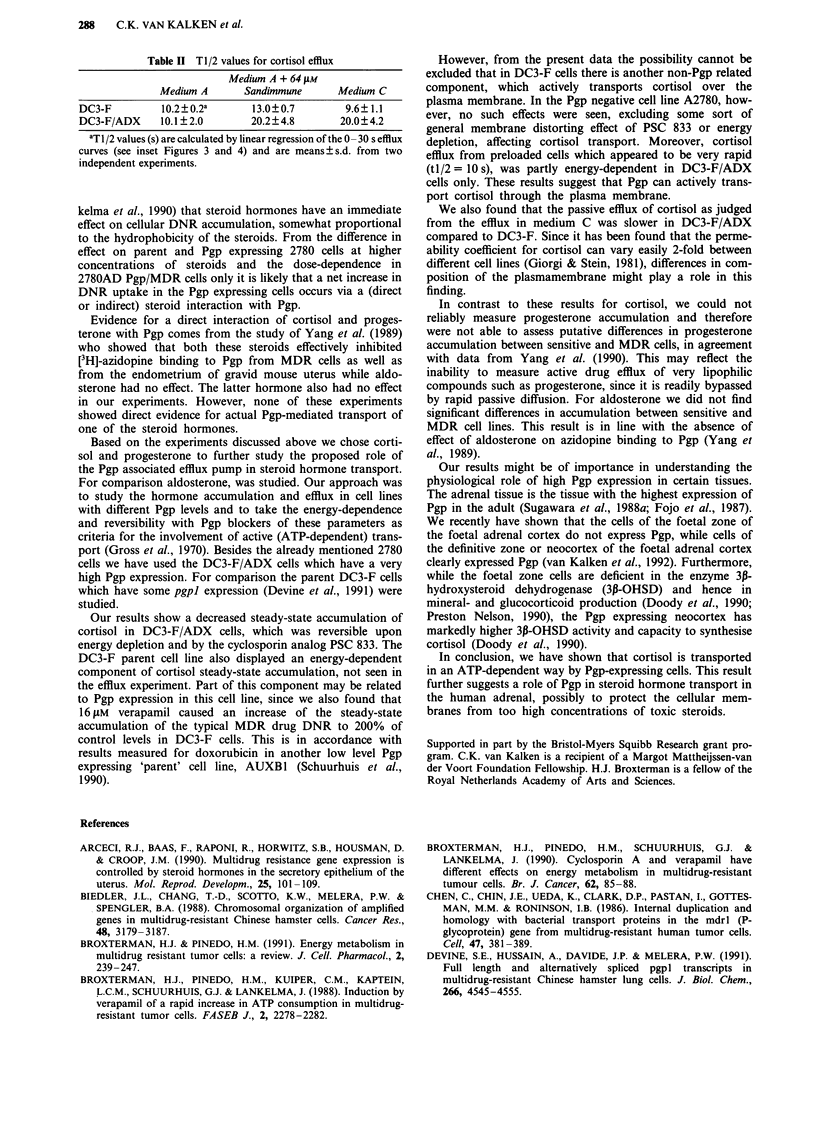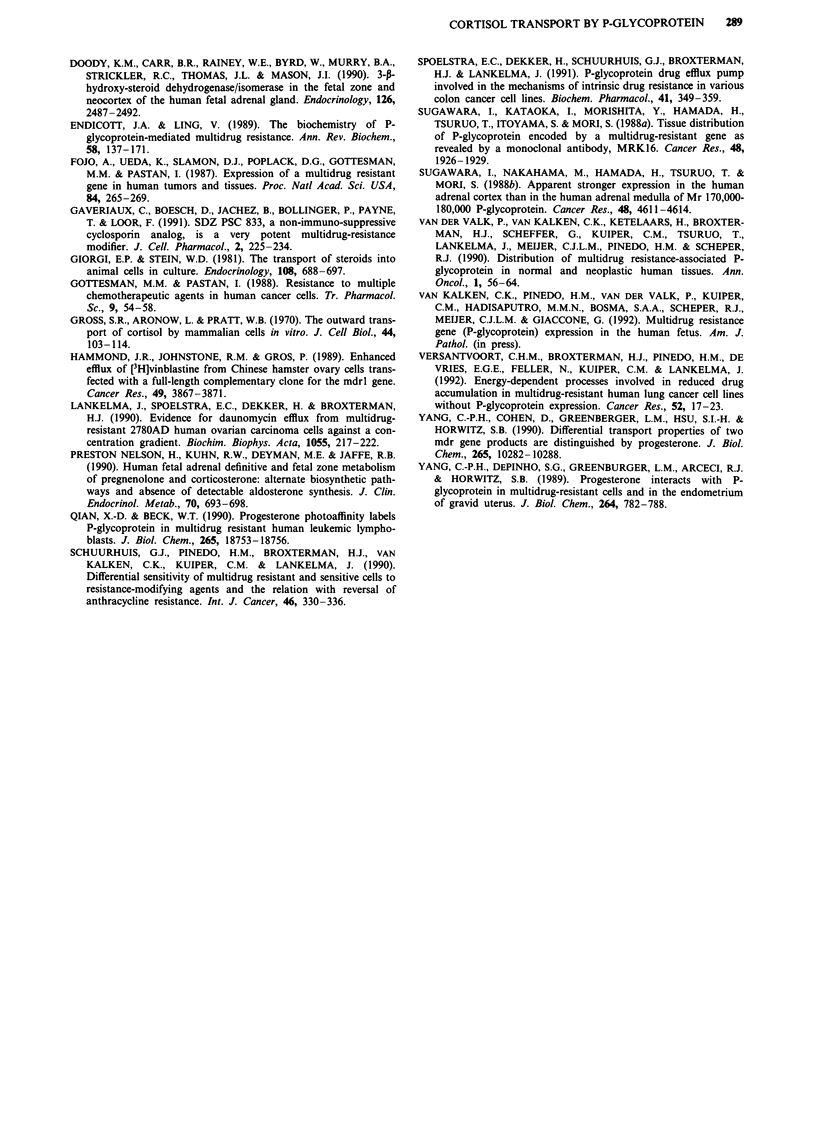# Cortisol is transported by the multidrug resistance gene product P-glycoprotein.

**DOI:** 10.1038/bjc.1993.54

**Published:** 1993-02

**Authors:** C. K. van Kalken, H. J. Broxterman, H. M. Pinedo, N. Feller, H. Dekker, J. Lankelma, G. Giaccone

**Affiliations:** Department of Medical Oncology, Free University Hospital, Amsterdam, The Netherlands.

## Abstract

The physiology of the multidrug transporter P-glycoprotein (Pgp) is still poorly understood. We now show evidence that cell lines with a high expression of Pgp display a reduced accumulation of cortisol and an ATP-dependent outward transport of the hormone. Cortisol efflux from Pgp negative cells does not have such an active component. Further we show that the steroid hormones cortisol, testosterone, and progesterone cause an immediate, dose-dependent increase of daunorubicin accumulation in Pgp overexpressing cells. These effects are particularly apparent for the more lipophilic steroids. These results demonstrate that Pgp may function as a transporter for cortisol and suggest a physiological role of the protein in steroid handling by organs such as the adrenal.


Br. J. Cancer (1993), 67, 284–289 © Macmillan Press Ltd., 1993

### Cortisol is transported by the multidrug resistance gene product P-glycoprotein

C.K. van Kalken[1], H.J. Broxterman[1], H.M. Pinedo[2], N. Feller[1], H. Dekker[1], J. Lankelma[1] & G. Giaccone[1]

[1]*Department of Medical Oncology, Free University Hospital and* [2]*Netherlands Cancer Institute, Amsterdam, The Netherlands.*

**Summary** The physiology of the multidrug transporter P-glycoprotein (Pgp) is still poorly understood. We now show evidence that cell lines with a high expression of Pgp display a reduced accumulation of cortisol and an ATP-dependent outward transport of the hormone. Cortisol efflux from Pgp negative cells does not have such an active component. Further we show that the steroid hormones cortisol, testosterone, and progesterone cause an immediate, dose-dependent increase of daunorubicin accumulation in Pgp overexpressing cells. These effects are particularly apparent for the more lipophilic steroids.

These results demonstrate that Pgp may function as a transporter for cortisol and suggest a physiological role of the protein in steroid handling by organs such as the adrenal.

P-glycoprotein (Pgp), encoded by the *mdr1* gene is an integral membrane protein, that functions as an ATP hydrolysis-dependent transporter with broad specificity of certain anticancer drugs out of tumour cells (Endicott & Ling, 1989; Broxterman *et al.*, 1988; Chen *et al.*, 1986). The increased drug efflux caused by overexpression of Pgp leads to a decreased steady-state drug accumulation in tumour cells, making them resistant to the drugs involved (Hammond *et al.*, 1989). This type of resistance to multiple drugs is called multidrug resistance (MDR). High expression of Pgp has also been found in a number of normal tissues. The peculiar tissue distribution with elevated levels of Pgp in liver, kidney, jejunum and colon, suggests a role of Pgp in active transport of hydrophobic compounds originating from the diet into bile, urine and directly into the gastrointestinal tract (Gottesman & Pastan, 1988; Van der Valk *et al.*, 1990). Interestingly, Pgp is also highly expressed in hormone secreting organs, like the adrenal gland (Gottesman & Pastan, 1988), the trophoblast of the human placenta (Sugawara *et al.*, 1988*a*) and is induced in the uterine secretory epithelium of the mouse by a combination of estrogen and progesterone (Arceci *et al.*, 1990), which suggests a role of Pgp during pregnancy. Further indications for a specialised role in the physiology of steroid hormones come from the observation of a strong Pgp expression in the human adrenal cortex, but weak or no staining in the adrenal medulla (Van der Valk *et al.*, 1990; Sugawara *et al.*, 1988*b*).

It has been hypothesised that steroids might be transported by Pgp out of the adrenal cortical cells and also concentrated again in specific tissues such as in the endometrium by Pgp expressing luminal plasma membranes. In support of an interaction of progesterone and cortisol with Pgp it has been shown that these steroids inhibit [$^3$H]-azidopine photoaffinity labeling of Pgp (Yang *et al.*, 1989) and that progesterone itself can specifically bind to Pgp (Qian *et al.*, 1990). In addition progesterone has been shown to impair Pgp mediated vinblastine efflux from murine MDR cells (Yang *et al.*, 1990). In the latter study (Yang *et al.*, 1990) progesterone itself could not be shown to be transported by Pgp.

Here we report studies on the interaction of the less hydrophobic steroid hormone cortisol with Pgp and provide evidence for active cellular efflux of this hormone in cells hyperexpressing Pgp.

Correspondence: H.J. Broxterman, Department of Medical Oncology, BR 2.32, Free University Hospital, De Boelelaan 1117, 1081 HV Amsterdam, The Netherlands.
Received 26 June 1992; and in revised form 28 August 1992.

### Methods

### *Cell culture*

DC-3F transformed hamster lung cells and the highly drug resistant DC-3F/ADX derivative (gift from Dr J.L. Biedler) and A2780 human ovarian carcinoma cells and the drug resistant variant 2780AD (gift from Dr R.F. Ozols) were grown in Eagle's modified Dulbecco's minimal essential medium, buffered with bicarbonate and 20 mM Hepes and supplemented with 7.5% FCS (Gibco Europe, Paisley, Scotland) in a humidified atmosphere with 5–6% $CO_2$ at 37°C and subcultured by short trypsinisation upon reaching confluence (Broxterman *et al.*, 1990). Drug-resistant cells were cultured in drug-free medium for 48 h before the experiments.

### *Chemicals*

[1,2-$^3$H(N)]-cortisol (49.1 Ci mmol$^{-1}$) was obtained from NEN-Du Pont (Dreiech, Germany). Unlabelled steroids (Sigma, St. Louis, MO; Merck, Darmstadt, Germany) were dissolved in absolute ethanol (10 mM) and stored at 4°C. Cyclosporin A (in the formulation of Sandimmune$^R$) and SDZ PSC 833 were a gift of Sandoz BV (Uden, The Netherlands). PSC 833, ([3′-keto-Bmt$^1$]-[Val$^2$]-cyclosporin), which is a very potent MDR reversing agent (Gaveriaux *et al.*, 1991) was dissolved in absolute ethanol. Daunorubicin.HCl (Cerubidin$^R$) was from Rhône-Poulenc (Amstleveen, The Netherlands) and verapamil.HCl from Sigma (St. Louis, MO).

### *Cellular cortisol accumulation and efflux*

Cells ($2 \times 10^6$ cells ml$^{-1}$) were incubated with 1 μM $^3$H-cortisol at 37°C in medium A (growth medium buffered with 20 mM Hepes, pH = 7.4, supplemented with 10% FCS) or medium C (medium A without glucose, with added 10 mM sodium azide and 1 mg ml$^{-1}$ 2-deoxy-d-glucose). By incubation in medium C, cellular ATP is depleted to 10–15% of initial concentration within 15 min (Versantvoort *et al.*, 1992). After incubation, the cells were washed twice with ice-cold medium and then transferred to liquid scintillation fluid and radioactivity was counted.

For cortisol efflux studies, the cells were first incubated with 1 μM $^3$H-cortisol for 30 min in medium C (pH = 7.4) at 37°C, pelleted and rapidly washed once with fresh, ice-cold medium. Then the cells were resuspended in 1 ml cold medium and quadruplicate samples were taken to determine the zero-time values for the efflux curves. Seven hundred and fifty μl of the cell suspension was then added to 6.75 ml efflux medium of 37°C to start the cortisol efflux. Every 5 s a sample was pipetted out in cold wash medium and pelleted immediately. Cell-associated $^3$H-cortisol was measured subsequently after transfer of the cells to liquid scintillation fluid. To examine the effects of Pgp blocking agents on cortisol efflux, cells were loaded with medium A + 64 μM cyclosporin A (Sandimmune) and effluxed in the same medium. In preliminary experiments we found that 64 μM cyclosporin A or 8 μM PSC 388 readily inhibited Pgp mediated daunorubicin (DNR) efflux from DC3-F/ADX cells (not shown).

*Effect of steroids on daunorubicin accumulation*

The effect of steroid hormones on cellular DNR accumulation was measured in a dynamic way in a flow-through system, as described earlier (Lankelma *et al.*, 1990; Spoelstra *et al.*, 1991). In this system 5–10 × $10^6$ 2780AD MDR or A2780 drug sensitive cells were allowed to attach to the glass bottom plate overnight under normal growth conditions. An HPLC pump, equipped for micro-liquid chromatography (Gilson, Villiers-Le-Bel, France) was used to pump DNR containing medium A with 5% FCS over the monolayer of cells. DNR fluorescence in the medium was continuously monitored at the outlet of the flow-through system by a fluorescence monitor (model 3000, Perkin-Elmer, Norwalk, CT) at excitation/emission wavelengths of 480/560 nm. The cells were allowed to equilibrate until a steady-state signal of DNR was reached. Then a series of six pulses of the studied steroid was injected every 30 s into the flowing medium (flow rate 200 μl min$^{-1}$) via an HPLC injection valve, resulting in concentrations present above the cells as indicated. In Figures 1 and 2 the estimated concentration of steroid at the outlet of the flow-cell is given. The starting concentration at the inlet is about three times higher. In the same way, injections of verapamil, resulting in a concentration of approximately 25– 75 μM verapamil in the medium overflowing the cells, known to exert maximal effects in this system on DNR accumulation in 2780AD cells (Spoelstra *et al.*, 1991), were used as a positive control MDR modifier. An increase of DNR accumulation in the cells causes a concomitant decrease in extracellular DNR fluorescence, represented by a dip in the steady-state level seen by fluorescence monitoring at the outlet of the flow-through system. The increase in extracellular DNR after the initial dip as seen in MDR cells (Figures 2 and 3) reflects an increased efflux of DNR after disappearance of the modifying drug or hormone.

### Results

A number of steroids synthesised along the metabolic pathways in the human adrenal were studied. The immediate effects of these steroids on DNR accumulation in 2780AD cells were monitored in a flow-through system to study their interaction with Pgp. In 2780AD cells an increase in intracellular DNR concentration was observed for all steroids studied except for aldosterone. However, both depth and shape of the time-curves varied considerably among the steroid hormones. Time-curves representative for the different shapes observed are shown in Figure 1.

A qualitative correlation was found between the lipophilicity of the steroids and the depth of the dip observed after their injection. The latter parameter can be seen as a relative measure of immediate DNR pumping inhibition. The order of lipophilicity of the most important steroids studied here is progesterone > testosterone > cortisol > aldosterone. The octanol/water partition coefficient for these steroids are 124, 61, 12.6 and not studied resp., according to Giorgi and Stein (1981).

The largerst effects were observed for the aldosterone precursors progesterone, corticosterone and 11-deoxy-corticosterone and for the cortisol precursor 11-deoxy-cortisol. Intermediate effects were observed for 17-hydroxy-progester-

**Figure 1** Effect of verapamil and several steroid hormones on steady-state daunorubicin accumulation in 2780 cells as recorded in the flow-through experiment. Representative time-curves after pulse-injections (arrows) of hormones are shown. Verapamil concentration is approximately 25–75 μM, hormone concentrations 50–150 μM, as explained in the Methods section.

**Figure 2** Effect of increasing concentrations of progesterone on steady-state daunorubicin accumulation in 2780 cells as measured in the flow-through experiment. A representative experiment is shown. Progesterone concentrations after pulse-injections (arrows) indicated are estimated concentrations at the outlet of the flow-through system, as explained in the Methods sections.

**Figure 3** Efflux of cellular cortisol after loading DC3-F cells with 1 μM cortisol (set at 100%) and incubation in cortisol-free medium for the indicated times. Efflux in medium A (■—■), medium C (▲—▲) or medium A + 64 μM cyclosporin (◆—◆). Data are from a representative experiment; inset: linear regression curves. T1/2 values are in Table II.

one and for the androgens dehydroepiandrosterone, androstenedione and testosterone, while cortisol and cortisone showed small effects. Aldosterone showed no effect at all. Small effects of lower concentrations of the steroids on cellular DNR uptake, also correlating with lipophilicity were observed in drug sensitive A2780 cells (Figure 1). However, further increasing the concentration of the steroids did not result in an increase of the effect in A2780 cells, as is shown for progesterone in Figure 2, whereas in 2780AD cells a dose-dependent effect was observed.

Subsequently we have studied the interaction of some of the steroid hormones with Pgp by measuring their accumulation in sensitive and Pgp/MDR cells. First the steroids with extreme polarity values, aldosterone and progesterone were studied. We could not reliably measure cellular accumulation of progesterone as reported by Yang *et al.* (1990), who could not obtain evidence for Pgp mediated progesterone efflux, possibly because such an extremely hydrophobic compound has a rapid passive diffusion rate across the plasma membrane. For aldosterone no significant difference in cellular accumulation between sensitive and Pgp/MDR cells was found (not shown). Another steroid hormone, that might be actively transported by Pgp is cortisol, because it is more hydrophilic than progesterone, but less hydrophilic than aldosterone (Giorgi & Stein, 1981). We therefore studied the energy-dependence of cortisol transport in the highly Pgp expressing cell line, DC3-F/ADX (resistance factor for actinomycin D is 10,000; Biedler *et al.*, 1988). In preliminary experiments we determined that the steady-state accumulation of cortisol in parent and Pgp expressing cells was reached within 15 min, while similar results were obtained upon longer incubation times. The results (Table I) showed that cortisol accumulation in DC3-F/ADX cells was about 30% of that in the parent cells. 2780AD cells accumulated about 65% cortisol compared to the parental A2780 cells. For the typical MDR drug DNR these values were 15% (DNR accumulation in DC3-F was 102, and in DC3-F/ADX 15 pmol $10^{-6}$ cells, at 0.5 μM DNR concentration) and 9% (2780AD compared to A2780, Broxterman *et al.*, 1988).

Further the cortisol accumulation in DC3-F/ADX was highly increased (to 800%) by adding 8 μM SDZ PSC 833 or (to 750%) by depleting cellular ATP levels below the level needed for proper Pgp function (Broxterman & Pinedo, 1991). In a separate experiment it was found that 16 μM verapamil as well as 8 μM cyclosporin A increased cortisol steady-state accumulation with about 300%.

In DC3-F cells the cortisol accumulation increased 2-fold under these conditions. In 2780AD cells the cortisol accumulation was doubled with 8 μM PSC 833 or ATP depletion, while no effect was seen in A2780.

To study cortisol efflux we loaded DC3-F and DC3-F/ADX cells with cortisol in medium C or medium A + 64 μM cyclosporin A to obtain equal cellular steady-state levels of cortisol (see Table I). The retention of cortisol in the cells was then measured upon incubation in medium A, C, or A + 64 μM cyclosporin A. For the efflux experiments we used

**Table I** Steady-state cortisol accumulation

| | *Medium A* | *Medium A + 8 μM PSC833* | *Medium C* | *C:A × 100%* |
|---|---|---|---|---|
| DC-3F | 1.3±0.5[a] | 3.1±1.4 | 2.8±0.1 | = 215% |
| DC3-F/ADX | 0.4±0.2 | 3.3±0.5 | 3.0±0.0 | = 750% |
| A2780 | 3.1±0.5 | 3.0±0.2 | 2.7±0.1 | = 87% |
| 2780AD | 2.0±0.7 | 3.4±0.6 | 3.6±0.6 | = 180% |

[a]Results are expressed in pmol/$10^6$ cells and are means ± s.d. of 60 min cortisol accumulation values of 3–5 (medium A) or 2 (medium C) independent experiments (each in quadruplicate). Cortisol concentration was 1 μM.

the latter drug instead of PSC 833, because of a limited supply of PSC 833. A higher concentration of cyclosporin A is needed, because PSC 833 is about 10-fold more potent than cyclosporin A in reversing MDR (Gaveriaux *et al.*, 1991). In Figures 3 and 4 and Table II it is shown that cortisol efflux is slowed down by energy-depletion or by the presence of cyclosporin A in the efflux medium in DC3-F/ADX, but not in DC3-F cells. The t1/2 values of the cortisol elimination from the cells as determined from the initial part of the semilogarithmically plotted data (see insert Figures 3 and 4) are compiled in Table II. When the cells were loaded in medium A with 8 μM cortisol instead of medium C with 1 μM cortisol (both giving similar intracellular cortisol concentrations), the t1/2 values for cortisol efflux from DC3-F/ADX in medium A were still about 3-fold shorter (not shown). These very rapid efflux values can however not be determined with great precision. This result was to be expected because after loading in medium C it may take a few seconds in medium A for ATP levels to recover sufficiently to fully support Pgp mediated efflux. In accordance with this explanation, loading of DC3-F cells in medium A or C made no difference for the T1/2 of apparent cortisol efflux (not shown), again suggesting its passive nature.

### Discussion

Although a role of Pgp in the active transport of steroid hormones was postulated, there was no experimental evidence to support it sofar.

We have now shown in an on-line detection system (Lan-

**Figure 4** Efflux of cellular cortisol after loading DC3-F/ADX cells with 1 μM cortisol and incubation in cortisol-free medium. For details see legend Figure 3.

**Table II** T1/2 values for cortisol efflux

| | *Medium A* | *Medium A + 64 μM Sandimmune* | *Medium C* |
|---|---|---|---|
| DC3-F | 10.2±0.2[a] | 13.0±0.7 | 9.6±1.1 |
| DC3-F/ADX | 10.1±2.0 | 20.2±4.8 | 20.0±4.2 |

[a]T1/2 values (s) are calculated by linear regression of the 0–30 s efflux curves (see inset Figures 3 and 4) and are means±s.d. from two independent experiments.

kelma *et al.*, 1990) that steroid hormones have an immediate effect on cellular DNR accumulation, somewhat proportional to the hydrophobicity of the steroids. From the difference in effect on parent and Pgp expressing 2780 cells at higher concentrations of steroids and the dose-dependence in 2780AD Pgp/MDR cells only it is likely that a net increase in DNR uptake in the Pgp expressing cells occurs via a (direct or indirect) steroid interaction with Pgp.

Evidence for a direct interaction of cortisol and progesterone with Pgp comes from the study of Yang *et al.* (1989) who showed that both these steroids effectively inhibited [$^3$H]-azidopine binding to Pgp from MDR cells as well as from the endometrium of gravid mouse uterus while aldosterone had no effect. The latter hormone also had no effect in our experiments. However, none of these experiments showed direct evidence for actual Pgp-mediated transport of one of the steroid hormones.

Based on the experiments discussed above we chose cortisol and progesterone to further study the proposed role of the Pgp associated efflux pump in steroid hormone transport. For comparison aldosterone, was studied. Our approach was to study the hormone accumulation and efflux in cell lines with different Pgp levels and to take the energy-dependence and reversibility with Pgp blockers of these parameters as criteria for the involvement of active (ATP-dependent) transport (Gross *et al.*, 1970). Besides the already mentioned 2780 cells we have used the DC3-F/ADX cells which have a very high Pgp expression. For comparison the parent DC3-F cells which have some *pgp1* expression (Devine *et al.*, 1991) were studied.

Our results show a decreased steady-state accumulation of cortisol in DC3-F/ADX cells, which was reversible upon energy depletion and by the cyclosporin analog PSC 833. The DC3-F parent cell line also displayed an energy-dependent component of cortisol steady-state accumulation, not seen in the efflux experiment. Part of this component may be related to Pgp expression in this cell line, since we also found that 16 μM verapamil caused an increase of the steady-state accumulation of the typical MDR drug DNR to 200% of control levels in DC3-F cells. This is in accordance with results measured for doxorubicin in another low level Pgp expressing 'parent' cell line, AUXB1 (Schuurhuis *et al.*, 1990).

However, from the present data the possibility cannot be excluded that in DC3-F cells there is another non-Pgp related component, which actively transports cortisol over the plasma membrane. In the Pgp negative cell line A2780, however, no such effects were seen, excluding some sort of general membrane distorting effect of PSC 833 or energy depletion, affecting cortisol transport. Moreover, cortisol efflux from preloaded cells which appeared to be very rapid (t1/2 = 10 s), was partly energy-dependent in DC3-F/ADX cells only. These results suggest that Pgp can actively transport cortisol through the plasma membrane.

We also found that the passive efflux of cortisol as judged from the efflux in medium C was slower in DC3-F/ADX compared to DC3-F. Since it has been found that the permeability coefficient for cortisol can vary easily 2-fold between different cell lines (Giorgi & Stein, 1981), differences in composition of the plasmamembrane might play a role in this finding.

In contrast to these results for cortisol, we could not reliably measure progesterone accumulation and therefore were not able to assess putative differences in progesterone accumulation between sensitive and MDR cells, in agreement with data from Yang *et al.* (1990). This may reflect the inability to measure active drug efflux of very lipophilic compounds such as progesterone, since it is readily bypassed by rapid passive diffusion. For aldosterone we did not find significant differences in accumulation between sensitive and MDR cell lines. This result is in line with the absence of effect of aldosterone on azidopine binding to Pgp (Yang *et al.*, 1989).

Our results might be of importance in understanding the physiological role of high Pgp expression in certain tissues. The adrenal tissue is the tissue with the highest expression of Pgp in the adult (Sugawara *et al.*, 1988*a*; Fojo *et al.*, 1987). We recently have shown that the cells of the foetal zone of the foetal adrenal cortex do not express Pgp, while cells of the definitive zone or neocortex of the foetal adrenal cortex clearly expressed Pgp (van Kalken *et al.*, 1992). Furthermore, while the foetal zone cells are deficient in the enzyme 3β-hydroxysteroid dehydrogenase (3β-OHSD) and hence in mineral- and glucocorticoid production (Doody *et al.*, 1990; Preston Nelson, 1990), the Pgp expressing neocortex has markedly higher 3β-OHSD activity and capacity to synthesise cortisol (Doody *et al.*, 1990).

In conclusion, we have shown that cortisol is transported in an ATP-dependent way by Pgp-expressing cells. This result further suggests a role of Pgp in steroid hormone transport in the human adrenal, possibly to protect the cellular membranes from too high concentrations of toxic steroids.

Supported in part by the Bristol-Myers Squibb Research grant program. C.K. van Kalken is a recipient of a Margot Mattheijssen-van der Voort Foundation Fellowship. H.J. Broxterman is a fellow of the Royal Netherlands Academy of Arts and Sciences.

### References

ARCECI, R.J., BAAS, F., RAPONI, R., HORWITZ, S.B., HOUSMAN, D. & CROOP, J.M. (1990). Multidrug resistance gene expression is controlled by steroid hormones in the secretory epithelium of the uterus. *Mol. Reprod. Developm.*, **25**, 101–109.

BIEDLER, J.L., CHANG, T.-D., SCOTTO, K.W., MELERA, P.W. & SPENGLER, B.A. (1988). Chromosomal organization of amplified genes in multidrug-resistant Chinese hamster cells. *Cancer Res.*, **48**, 3179–3187.

BROXTERMAN, H.J. & PINEDO, H.M. (1991). Energy metabolism in multidrug resistant tumor cells: a review. *J. Cell. Pharmacol.*, **2**, 239–247.

BROXTERMAN, H.J., PINEDO, H.M., KUIPER, C.M., KAPTEIN, L.C.M., SCHUURHUIS, G.J. & LANKELMA, J. (1988). Induction by verapamil of a rapid increase in ATP consumption in multidrug-resistant tumor cells. *FASEB J.*, **2**, 2278–2282.

BROXTERMAN, H.J., PINEDO, H.M., SCHUURHUIS, G.J. & LANKELMA, J. (1990). Cyclosporin A and verapamil have different effects on energy metabolism in multidrug-resistant tumour cells. *Br. J. Cancer*, **62**, 85–88.

CHEN, C., CHIN, J.E., UEDA, K., CLARK, D.P., PASTAN, I., GOTTESMAN, M.M. & RONINSON, I.B. (1986). Internal duplication and homology with bacterial transport proteins in the mdr1 (P-glycoprotein) gene from multidrug-resistant human tumor cells. *Cell*, **47**, 381–389.

DEVINE, S.E., HUSSAIN, A., DAVIDE, J.P. & MELERA, P.W. (1991). Full length and alternatively spliced pgp1 transcripts in multidrug-resistant Chinese hamster lung cells. *J. Biol. Chem.*, **266**, 4545–4555.

DOODY, K.M., CARR, B.R., RAINEY, W.E., BYRD, W., MURRY, B.A., STRICKLER, R.C., THOMAS, J.L. & MASON, J.I. (1990). 3-β-hydroxy-steroid dehydrogenase/isomerase in the fetal zone and neocortex of the human fetal adrenal gland. *Endocrinology*, **126**, 2487–2492.

ENDICOTT, J.A. & LING, V. (1989). The biochemistry of P-glycoprotein-mediated multidrug resistance. *Ann. Rev. Biochem.*, **58**, 137–171.

FOJO, A., UEDA, K., SLAMON, D.J., POPLACK, D.G., GOTTESMAN, M.M. & PASTAN, I. (1987). Expression of a multidrug resistant gene in human tumors and tissues. *Proc. Natl Acad. Sci. USA*, **84**, 265–269.

GAVERIAUX, C., BOESCH, D., JACHEZ, B., BOLLINGER, P., PAYNE, T. & LOOR, F. (1991). SDZ PSC 833, a non-immuno-suppressive cyclosporin analog, is a very potent multidrug-resistance modifier. *J. Cell. Pharmacol.*, **2**, 225–234.

GIORGI, E.P. & STEIN, W.D. (1981). The transport of steroids into animal cells in culture. *Endocrinology*, **108**, 688–697.

GOTTESMAN, M.M. & PASTAN, I. (1988). Resistance to multiple chemotherapeutic agents in human cancer cells. *Tr. Pharmacol. Sc.*, **9**, 54–58.

GROSS, S.R., ARONOW, L. & PRATT, W.B. (1970). The outward transport of cortisol by mammalian cells *in vitro*. *J. Cell Biol.*, **44**, 103–114.

HAMMOND, J.R., JOHNSTONE, R.M. & GROS, P. (1989). Enhanced efflux of [$^3$H]vinblastine from Chinese hamster ovary cells transfected with a full-length complementary clone for the mdr1 gene. *Cancer Res.*, **49**, 3867–3871.

LANKELMA, J., SPOELSTRA, E.C., DEKKER, H. & BROXTERMAN, H.J. (1990). Evidence for daunomycin efflux from multidrug-resistant 2780AD human ovarian carcinoma cells against a concentration gradient. *Biochim. Biophys. Acta*, **1055**, 217–222.

PRESTON NELSON, H., KUHN, R.W., DEYMAN, M.E. & JAFFE, R.B. (1990). Human fetal adrenal definitive and fetal zone metabolism of pregnenolone and corticosterone: alternate biosynthetic pathways and absence of detectable aldosterone synthesis. *J. Clin. Endocrinol. Metab.*, **70**, 693–698.

QIAN, X.-D. & BECK, W.T. (1990). Progesterone photoaffinity labels P-glycoprotein in multidrug resistant human leukemic lymphoblasts. *J. Biol. Chem.*, **265**, 18753–18756.

SCHUURHUIS, G.J., PINEDO, H.M., BROXTERMAN, H.J., VAN KALKEN, C.K., KUIPER, C.M. & LANKELMA, J. (1990). Differential sensitivity of multidrug resistant and sensitive cells to resistance-modifying agents and the relation with reversal of anthracycline resistance. *Int. J. Cancer*, **46**, 330–336.

SPOELSTRA, E.C., DEKKER, H., SCHUURHUIS, G.J., BROXTERMAN, H.J. & LANKELMA, J. (1991). P-glycoprotein drug efflux pump involved in the mechanisms of intrinsic drug resistance in various colon cancer cell lines. *Biochem. Pharmacol.*, **41**, 349–359.

SUGAWARA, I., KATAOKA, I., MORISHITA, Y., HAMADA, H., TSURUO, T., ITOYAMA, S. & MORI, S. (1988*a*). Tissue distribution of P-glycoprotein encoded by a multidrug-resistant gene as revealed by a monoclonal antibody, MRK16. *Cancer Res.*, **48**, 1926–1929.

SUGAWARA, I., NAKAHAMA, M., HAMADA, H., TSURUO, T. & MORI, S. (1988*b*). Apparent stronger expression in the human adrenal cortex than in the human adrenal medulla of Mr 170,000-180,000 P-glycoprotein. *Cancer Res.*, **48**, 4611–4614.

VAN DER VALK, P., VAN KALKEN, C.K., KETELAARS, H., BROXTERMAN, H.J., SCHEFFER, G., KUIPER, C.M., TSURUO, T., LANKELMA, J., MEIJER, C.J.L.M., PINEDO, H.M. & SCHEPER, R.J. (1990). Distribution of multidrug resistance-associated P-glycoprotein in normal and neoplastic human tissues. *Ann. Oncol.*, **1**, 56–64.

VAN KALKEN, C.K., PINEDO, H.M., VAN DER VALK, P., KUIPER, C.M., HADISAPUTRO, M.M.N., BOSMA, S.A.A., SCHEPER, R.J., MEIJER, C.J.L.M. & GIACCONE, G. (1992). Multidrug resistance gene (P-glycoprotein) expression in the human fetus. *Am. J. Pathol.* (in press).

VERSANTVOORT, C.H.M., BROXTERMAN, H.J., PINEDO, H.M., DE VRIES, E.G.E., FELLER, N., KUIPER, C.M. & LANKELMA, J. (1992). Energy-dependent processes involved in reduced drug accumulation in multidrug-resistant human lung cancer cell lines without P-glycoprotein expression. *Cancer Res.*, **52**, 17–23.

YANG, C.-P.H., COHEN, D., GREENBERGER, L.M., HSU, S.I.-H. & HORWITZ, S.B. (1990). Differential transport properties of two mdr gene products are distinguished by progesterone. *J. Biol. Chem.*, **265**, 10282–10288.

YANG, C.-P.H., DEPINHO, S.G., GREENBURGER, L.M., ARCECI, R.J. & HORWITZ, S.B. (1989). Progesterone interacts with P-glycoprotein in multidrug-resistant cells and in the endometrium of gravid uterus. *J. Biol. Chem.*, **264**, 782–788.